# New Biomarkers in Autoimmune Disease

**DOI:** 10.1155/2017/8702425

**Published:** 2017-04-12

**Authors:** Guixiu Shi, Zhixin Zhang, Quanzhen Li

**Affiliations:** ^1^Department of Rheumatology and Clinical Immunology, The First Hospital of Xiamen University, Xiamen 361003, China; ^2^Veterans Affairs Nebraska-Western Iowa Health Care System, University of Nebraska Medical Center, Omaha, NE, USA; ^3^Department of Immunology, University of Texas Southwestern Medical Center, 5323 Harry Hines Blvd., Dallas, TX, USA

Autoimmune diseases are a group of diverse diseases with a common etiology in which the immune system responds to self-antigens leading to damage or dysfunction of tissues. Biomarkers are those measurable substances in the body which associate with the intensity of a disease or other physiological states. Biomarkers can be used in many fields of clinical practice such as disease diagnosis, disease activity evaluation, and disease pathogenesis study. Biomarker research is a rapidly advancing area in autoimmune disease. A variety of researches on discovering new biomarkers including autoantibodies in autoimmune diseases have been conducted in recent years.

Based on this background, this special issue presents recent advances for new biomarkers in autoimmune diseases. From 17 submissions, 8 papers are published covering these autoimmune diseases including systemic lupus erythematosus (SLE), lupus nephritis (LN), polymyositis, and ankylosing spondylitis (AS).

SLE is one of the classical autoimmune diseases characterized by a wide profile of autoantibodies. Z. Da et al. in their article “CXCL13 Promotes Proliferation of Mesangial Cells by Combination with CXCR5 in SLE” found CXCL13 was overexpressed in SLE especially in lupus nephritis patients, it promoted the proliferation of a human renal mesangial cell, and the effect was weakened after the silence of CXCR5. CXCL13-CXCR5 axis is suggested as a new therapeutic target in LN. In another research, De N. He et al. in their article “Association of Serum CXCL13 with Intrarenal Ectopic Lymphoid Tissue Formation in Lupus Nephritis” discovered increased serum levels of CXCL13 play an important role in the formation of renal ectopic lymphoid tissue and the pathological renal impairment process in LN and suggested CXCL13 and the formation of ELT in renal tissues might be an important therapy target for LN. J. Xue et al. in their article “Dickkopf-1 Is a Biomarker for Systemic Lupus Erythematosus and Active Lupus Nephritis” compared the concentrations of Wnt-3A, Frizzled-8, and Dickkopf-1 (DKK-1) of Wnt signaling in SLE patients (with or without LN) and healthy cohorts. The result demonstrated that the serum DKK-1 was considered a better positive biomarker for identification of LN in SLE patients. Therefore, the serum and/or urine DKK-1 may be a valuable and independent biomarker for identification of SLE patients with active LN. R. Bai et al. in their article “Depressive and Anxiety Disorders in Systemic Lupus Erythematosus Patients without Major Neuropsychiatric Manifestations” observed high prevalence of depression and anxiety in SLE patients without major neuropsychiatric manifestations, and anxiety was associated with anti-P0 antibody. S. Li et al. in their article “Link-Polymorphism of 5-HTT Promoter Region Is Associated with Autoantibodies in Patients with Systemic Lupus Erythematosus” found the frequency of SS genotype and S allele of serotonin transporter-linked polymorphic region (5-HTTLPR) in SLE patients with positive anti-Sm antibody and anti-U1RNP antibody significantly higher than the other genotypes and allele and speculated 5-HTTLPR affecting production of some autoantibodies, especially anti-Sm and anti-U1RNP antibody in SLE.

Ankylosing spondylitis is the prototype of immune-mediated inflammatory rheumatic arthritis. C. Wang et al. in their article “Serum HMGB1 Serves as a Novel Laboratory Indicator Reflecting Disease Activity and Treatment Response in Ankylosing Spondylitis Patients” analyzed the association between serum levels of HMGB1 and clinical features of AS patients before and during treatment. Serum level of HMGB1 was higher in AS patients. It reflects the disease activity of AS and serves as a laboratory indicator for the therapeutic response.

The dysfunction of a peripheral blood mononuclear cell (PBMC) is involved in autoimmune diseases. Q. Xie et al. in their article “DEPTOR-mTOR Signaling Is Critical for Lipid Metabolism and Inflammation Homeostasis of Lymphocytes in Human PBMC Culture” reported combination of DEP domain-containing mTOR-interacting protein (DEPTOR) overexpression and mTORC2/AKT inhibitors effectively inhibits lipogenesis and inflammation in lymphocytes of PBMC culture, implying DEPTOR-mTOR in lymphocytes of PBMC as a biomarker for detecting and treating autoimmune diseases. G. Yin et al. in their article “Identification of Palmitoleic Acid Controlled by mTOR Signaling as a Biomarker of Polymyositis” confirmed the increased palmitoleic acid level in PBMC of patients with polymyositis, and it bears the potential to be a new marker of abnormal PBMC in polymyositis.

These papers above represent some interesting, novel advance in a biomarker for autoimmune diseases. We hope this special issue would provide some new ideas for diagnosis and treatment.

